# Generation of hydroxyl radicals from reactions between a dimethoxyhydroquinone and iron oxide nanoparticles

**DOI:** 10.1038/s41598-018-29075-5

**Published:** 2018-07-17

**Authors:** Gry Lyngsie, Lelde Krumina, Anders Tunlid, Per Persson

**Affiliations:** 1 Center of Environmental and Climate Research, Lund University, SE-223 62 Lund, Sweden; 2 Department of Biology, Lund University, SE-223 62 Lund, Sweden

## Abstract

The hydroxyl radical (·OH) is a powerful oxidant that is produced in a wide range of environments via the Fenton reaction (Fe^2+^  + H_2_O_2_ → Fe^3+^  + ·OH + OH^-^). The reactants are formed from the reduction of Fe^3+^ and O_2_, which may be promoted by organic reductants, such as hydroquinones. The aim of this study was to investigate the extent of ·OH formation in reactions between 2,6-dimethoxyhydroquinone (2,6-DMHQ) and iron oxide nanoparticles. We further compared the reactivities of ferrihydrite and goethite and investigated the effects of the O_2_ concentration and pH on the generation of ·OH. The main finding was that the reactions between 2,6-DMHQ and iron oxide nanoparticles generated substantial amounts of ·OH under certain conditions via parallel reductive dissolution and catalytic oxidation reactions. The presence of O_2_ was essential for the catalytic oxidation of 2,6-DMHQ and the generation of H_2_O_2_. Moreover, the higher reduction potential of ferrihydrite relative to that of goethite made the former species more susceptible to reductive dissolution, which favored the production of ·OH. The results highlighted the effects of surface charge and ligand competition on the 2,6-DMHQ oxidation processes and showed that the co-adsorption of anions can promote the generation of ·OH.

## Introduction

The hydroxyl radical (·OH) is a powerful oxidant that is produced in a wide range of environments^1^. This radical degrades organic compounds^1–3^, which makes it harmful if produced in excess and in close proximity to cells. Such oxidative stress reactions can have a range of adverse effects, and ·OH production may be accelerated by reactive nanoparticles^4^. The oxidative power of ·OH can also be favourably exploited, for instance in pollutant and wastewater treatments. Soil remediation studies have demonstrated that belowground injection of hydrogen peroxide (H_2_O_2_) effectively degrades organic contaminants, particularly in the presence of iron minerals^5–8^. Similar processes occur in terrestrial systems without human interference and have been suggested to play an important role in the degradation of natural organic matter, thereby contributing to the terrestrial carbon cycle^9–14^.

The Fenton reaction

1 $${{\rm{Fe}}}^{2+}+{{\rm{H}}}_{2}{{\rm{O}}}_{2}\to {{\rm{Fe}}}^{3+}+\cdot \,{\rm{OH}}+{{\rm{OH}}}^{-}$$

is an important source of ·OH, and to trigger this reaction, Fe^2+^ and H_2_O_2_ must co-exist. In homogeneous solutions, the generation of ·OH is strongly pH dependent. At very acidic pH values, H_2_O_2_ is stabilized as H_3_O_2_^+^, which is deprotonated and subsequently disproportionates into O_2_ and H_2_O with an increase in the pH^3^. A pH increase also promotes the oxidation of Fe^2+^ ^15^, and therefore, the Fenton reaction has an optimum pH between 3 and 4^2^. The regeneration of Fe^2+^ is accomplished either by oxygen species (e.g., the Haber-Weiss mechanism) or by organic reductants. H_2_O_2_ is produced via the enzymatic reduction of O_2_ or by redox-active metabolites^10,16^. In this respect, hydroquinones are an interesting class of organic metabolites because they can reduce both O_2_ and Fe^3+^, thereby generating both Fenton reagents^17–19^. In fact, certain fungi have evolved wood-degrading machinery that partially depends on the ·OH generated from reactions between 2,5-dimethoxy-1,4-hydroquinone (2,5-DMHQ (1,4-(CH_3_O)_2_-2,5-C_6_H_2_(OH)_2_)) and Fe^3+^ ^9,11,13,16^.

In oxic environments, the reductive dissolution of Fe(III) oxides may provide the only source of Fe^2+^, which affects the pH dependence and mechanism of ·OH generation^20–24^. Moreover, reactive Fe(III) oxide nanoparticles can provide catalytically active surfaces for H_2_O_2_ generation and degradation^2,25^. This feature makes the reactions between such nanoparticles and redox-active metabolites particularly interesting because of the possibility of forming Fenton reagents at the particle surfaces, which increases the likelihood that molecules accumulated at these interfaces will be oxidized. Recently, reactions between 2,6-dimethoxy-1,4-hydroquinone (2,6-DMHQ (1,4-(CH_3_O)_2_-2,6-C_6_H_2_(OH)_2_)) and iron oxides were shown to cause reductive dissolution as well as catalytic hydroquinone oxidation in the presence of O_2_^26^, but whether these reactions also generate significant amounts of ·OH remains unknown.

The detection and quantification of the short-lived ·OH species (lifetimes of ca. 10^−3^ to 10^−6^ s in aqueous solutions)^27,28^ are challenging, and often, ·OH has to be captured and measured with a probe molecule. Recent results showed that terephthalic acid (TPA (C_6_H_4_-1,4-(CO_2_H)_2_)) is a suitable probe for ·OH detection;^29^ thus, this probe was used to address the main question of this study: to what extent is ·OH formed by reactions between a hydroquinone (2,6-DMHQ) and iron oxide nanoparticles? The reactivity of ferrihydrite and goethite nanoparticles was further compared, and the influence of the O_2_ level and pH was investigated. The hydroxylated probe (hTPA(C_6_H_4_-1,4-(CO_2_H)_2_-2-OH)) has been suggested^30–32^ but not shown to adsorb to surfaces below a neutral pH. Thus, the effect of probe adsorption is also addressed herein.

## Results and Discussion

The addition of any probe, be it a fluorescence or spin-trap probe, or buffer has the potential to modify the redox reactions that occur at the iron oxide surface and thus affect the generation of ·OH. We begin, therefore, by discussing the adsorption of the probe molecules and compare the 2,6-DMHQ-iron oxide redox reactions under aerobic and anaerobic conditions in the absence and presence of TPA; these conditions are characterized by initial dissolved oxygen concentrations of 270–310 and 0–20 μM, respectively (see Methods section).

### Interactions between the TPA/hTPA probe molecules and the iron oxide surface

**Figure 1:**
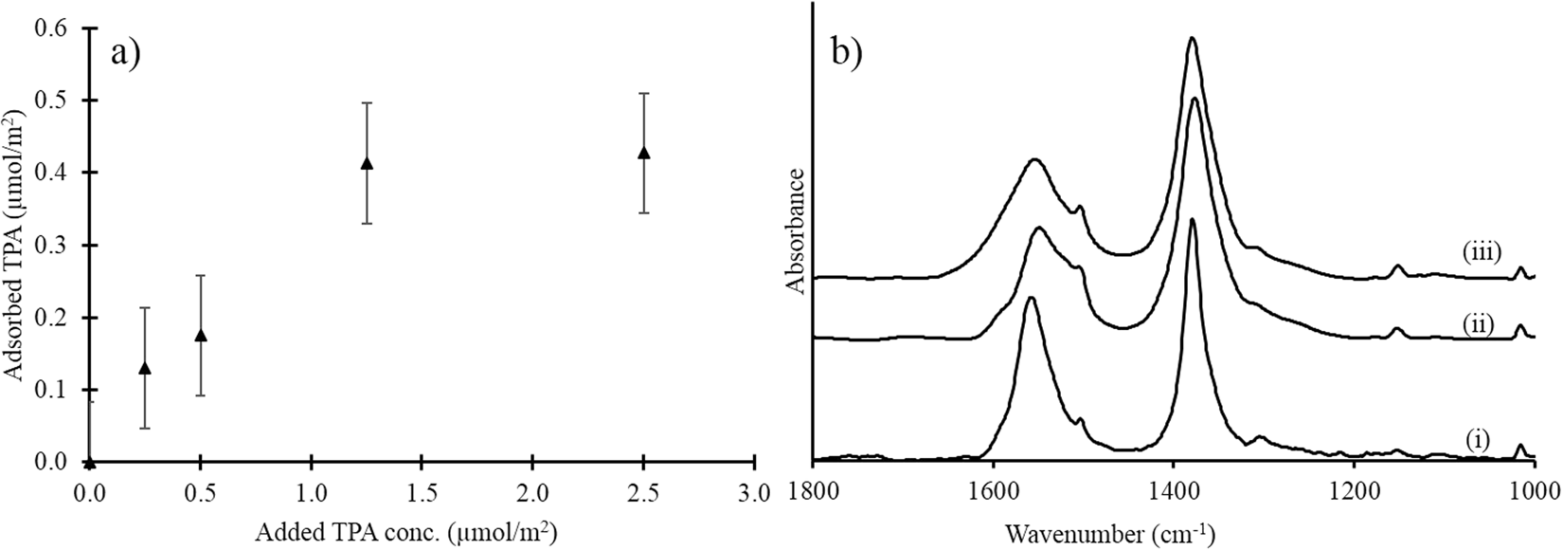
(**a**) Adsorption isotherm of TPA on ferrihydrite at pH 4.5 in 0.1 M NaCl. The reaction time was 60 minutes. 1 (**b**) IR spectra of (i) 10 mM TPA in an aqueous solution at pH 4.5, (ii) TPA adsorbed onto ferrihydrite at pH 4.5 with a surface coverage of 0.4 µmol/m^2^, and (iii) TPA adsorbed onto goethite at pH 5.0 with a surface coverage of 0.6 µmol/m^2^. The error bars are standard deviations of triplicate measurements.

The dicarboxylic structure of the TPA probe molecule indicates that it should have a strong affinity for iron oxide surfaces^33–37^. Indeed, a previous study examined the adsorption of TPA on goethite, and the maximum surface coverage (Γ_max_) was determined to be 0.5–0.6 µmol/m^2^ at pH 5.0^38^. This result is in good agreement with the adsorption onto ferrihydrite at pH 4.5 that was determined in the current study (Γ_max_ ∼ 0.5 µmol/m^2^, Fig. 1a) and indicates that the surface saturation is rather low relative to that of strongly adsorbing ions, such as phosphate and arsenate, whose surface saturation on iron oxides typically reaches approximately 2.0–2.5 µmol/m^2^ ^39,40^. Moreover, previous IR data suggest that TPA adsorption on goethite occurs via outer-sphere interactions^38^, i.e., TPA adsorbs via either surface hydration-shared ion pairs or solvent-surface hydration-separated ion pairs^41^. In the former class, the ligands are hydrogen-bonded to water molecules or hydroxyl groups in the surface layer, while in the latter class, the ligands retain the first solvation shell, and thus, at least one water molecule separates the ligands from the surface layer. The similarity between the IR spectra of the deprotonated TPA in solution and the spectra of TPA adsorbed onto ferrihydrite and goethite indicates that deprotonated outer-sphere complexes predominate on both iron oxides (Fig. 1b), although we cannot distinguish different outer-sphere species based on these data. Nevertheless, we can conclude that at the total TPA concentration (1.5 µmol/m^2^) used in the 2,6-DMHQ-iron oxide experiments, the surfaces are saturated with probe molecules, which exist predominantly as outer-sphere surface species. Hence, TPA should be in close proximity to any radical-generating processes that occur at the water-iron oxide interface.

**Figure 2:**
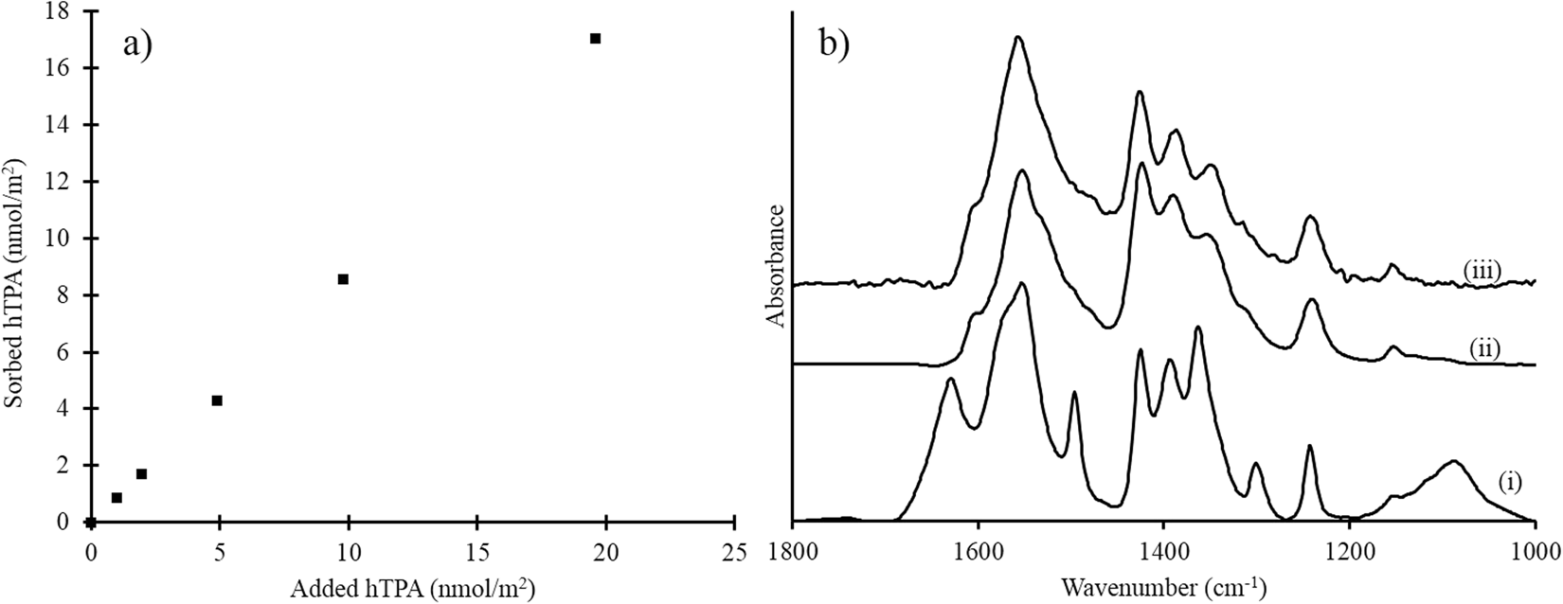
(**a**) Adsorption isotherm of hTPA on ferrihydrite at pH 4.5 in 0.1 M NaCl. The reaction time was 60 minutes. (**b**) IR spectra of (i) 20 mM hTPA in an aqueous solution at pH 4.5, (ii) hTPA adsorbed onto ferrihydrite at pH 4.5 with a surface coverage of 84 nmol/m^2^, and (iii) hTPA adsorbed onto goethite at pH 4.5 with a surface coverage of 75 nmol/m^2^. The error bars are the standard deviations of triplicate measurements, but in these experiments, the bars are smaller than the size of the symbols and are therefore not distinguishable.

The oxidized probe, hTPA, also adsorbed onto the iron oxides (Fig. 2). In this case, the IR spectra of the adsorbed hTPA were distinctly different from that of the aqueous counterpart (Fig. 2b), indicating direct coordination of the probe to the Fe^3+^ at the surface, i.e., inner-sphere surface coordination. This type of coordination was expected since a hydroxyl group was introduced at the position ortho to one of the carboxyl groups, yielding a structural fragment similar to salicylate, which has been shown to adsorb to metal oxide surfaces through inner-sphere interactions^42,43^. The inner-sphere coordination mode was facilitated by the formation of six-membered chelate structures. To determine the total amount of oxidized probe, this fraction of adsorbed hTPA must be desorbed (see SI and Fig. S3).

### Effects of the TPA probe on the 2,6-DMHQ-iron oxide redox reactions

As mentioned above, hydroquinones are oxidized by iron oxide surfaces via two main reaction pathways, namely, reductive dissolution (2) and surface catalysis (3):

2 $$2{\rm{FeOOH}}+{{\rm{H}}}_{2}{\rm{Q}}\to 2{{\rm{Fe}}}^{2+}+{\rm{Q}}+4{{\rm{OH}}}^{-}$$

3 $${{\rm{H}}}_{2}{\rm{Q}}+{{\rm{O}}}_{2}\mathop{\longrightarrow }\limits^{\begin{array}{c}{FeOOH}\\ {catalyst}\end{array}}\,{\rm{Q}}+{{\rm{H}}}_{2}{{\rm{O}}}_{2}$$

**Figure 3:**
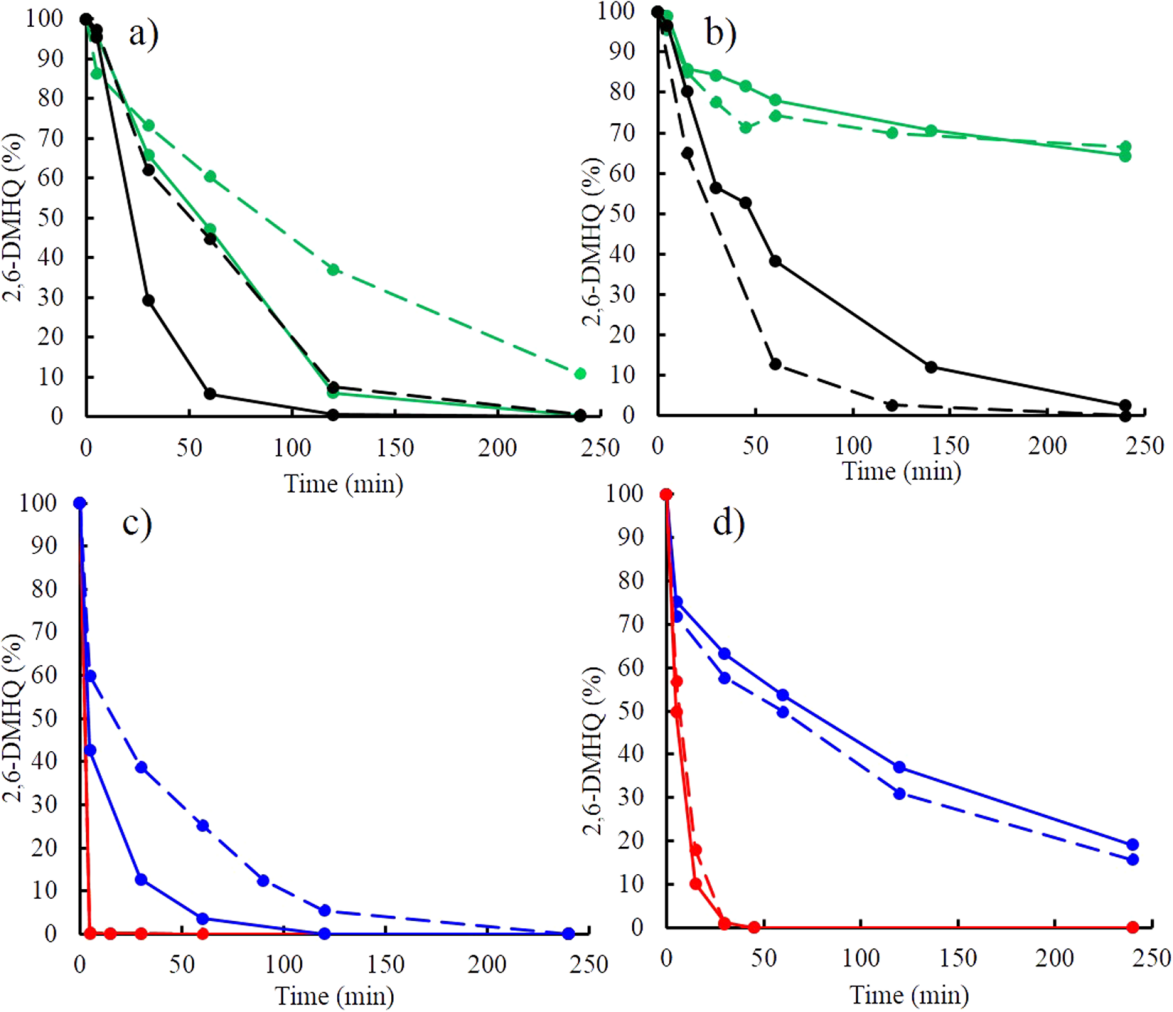
Oxidation of 90 µM 2,6-DMHQ (0.44 µmol/m^2^) at pH 4.5 (black: aerobic and green: anaerobic) and pH 7.0 (red: aerobic and blue: anaerobic) in the presence of ferrihydrite (**a**,**c**) and goethite (**b**,**d**). Solid lines represent the experiments conducted in the presence of 300 µM TPA, and the dashed lines represent those conducted in the absence of TPA.

Reactions (2) and (3) can be distinguished by comparing the oxidation reactions under aerobic and anaerobic conditions because surface catalysis is strongly O_2_-dependent while reductive dissolution is not. Our results showed that O_2_ had a large effect on 2,6-DMHQ oxidation, particularly in the presence of goethite (Fig. 3). At pH 4.5 under aerobic conditions, 100% of the hydroquinone was oxidized by goethite after 4 h, while only ca. 30% was oxidized anaerobically. The corresponding values in the ferrihydrite system under aerobic and anaerobic conditions were 100% and 90%, respectively. These results corroborate previous findings showing that goethite mainly mediated catalytic 2,6-DMHQ oxidation, whereas the reaction with ferrihydrite exhibited a large contribution from reductive dissolution, which was evidenced by the extensive 2,6-DMHQ oxidation observed under anaerobic conditions^26^.

The extent of aerobic 2,6-DMHQ oxidation increased further at pH 7.0; otherwise, similar differences between aerobic and anaerobic conditions as those at pH 4.5 were observed (Fig. 3c,d). Interestingly, substantial oxidation was also observed at pH 7.0 under anaerobic conditions. This oxidation is likely caused by reductive dissolution, which will be favoured if the Fe^2+^ concentration is maintained at low concentrations through re-oxidation by the low O_2_ concentrations that exists also under conditions where we tried to exclude oxygen^26^. In addition, these oxygen trace concentrations can also contribute to the catalytic oxidation of 2,6-DMHQ.

The O_2_ dependence and differences in reactivity between ferrihydrite and goethite were not dramatically affected by the presence of the TPA probe, although some changes in the extent of oxidation were observed (Fig. 3). TPA had an inhibiting effect on aerobic 2,6-DMHQ oxidation in the presence of goethite, which is in line with the occurrence of competitive reactions between the TPA anions and quinone species at the water-iron oxide interface. Since the catalytic pathway dominated aerobic oxidation, the results indicated that TPA interfered with the 2,6-DMHQ and/or O_2_ surface species involved in the catalytic process. Note that under anaerobic conditions, the 2,6-DMHQ oxidation by goethite in absence and presence of TPA was similar, which is consistent with the fact that both reductive dissolution and catalysis contribute to 2,6-DMHQ oxidation, as discussed in the paragraph above (Fig. 3b,d).

In contrast to the results for goethite, TPA increased the extent of 2,6-DMHQ oxidation by ferrihydrite under both aerobic and anaerobic conditions at pH 4.5 and 7.0 (Fig. 3a,c). The contribution of reductive dissolution was substantial in the presence of ferrihydrite, and thus, the TPA-promoted oxidation of 2,6-DMHQ is likely related to the reductive mechanism. The reason for this effect is not clear, but the outer-sphere adsorption of TPA on ferrihydrite will reduce the positive surface charge, which in turn will promote the adsorption of protons. Previously proposed mechanisms of reductive ferrihydrite dissolution suggest that protonation may affect the kinetics either via a preceding reaction step involving non-reductive dissolution or via surface reconstruction after Fe^2+^ dissolution^21^. Both scenarios could explain the increased extent of 2,6-DMHQ oxidation in the presence of TPA. Apparently, in the case of ferrihydrite, the relative contributions from reductive dissolution and catalytic oxidation were such that the rate-increasing effects induced by TPA out-weighed the competitive ones under aerobic conditions (Fig. 3a). Overall, the effects of TPA on 2,6-DMHQ oxidation further corroborated the differences between ferrihydrite and goethite.

**Figure 4:**
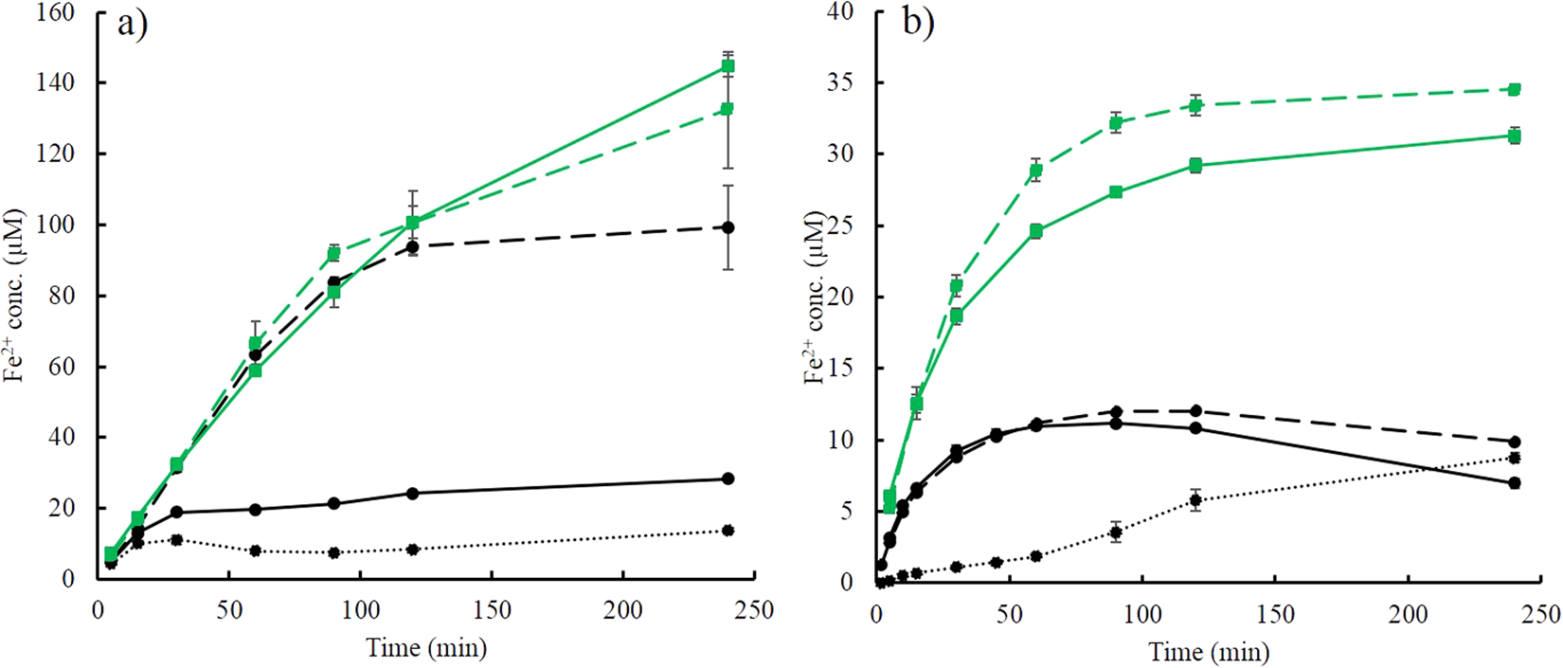
Generation of Fe^2+^ from reactions between 90 µM 2,6-DMHQ (0.44 µmol/m^2^) and (**a**) ferrihydrite and (**b**) goethite at pH 4.5. The black and green lines represent aerobic and anaerobic conditions, respectively. The experiments were carried out in the absence of TPA (dashed lines), in the presence of 300 µM TPA (solid lines), and in the presence of both 30 µM of H_2_O_2_ and 300 µM TPA (dotted lines). Note that the y-axis scales in (**a**) and (**b**) are different. The error bars are standard deviations of triplicate measurements. Some of these error bars are smaller than the size of the symbols and are therefore not distinguishable.

Aqueous Fe^2+^ is generated via the reductive dissolution of iron oxides caused by 2,6-DMHQ oxidation. It follows that the large difference in the contribution of this reaction resulted in a corresponding difference in the generation of Fe^2+^ from ferrihydrite and goethite (Fig. 4); note that no results were reported for the pH 7.0 experiments because the Fe^2+^ concentrations were below the limit of detection (LoD) of 2.0 μM. Under anaerobic conditions at pH 4.5, the Fe^2+^ concentration reached levels of approximately 120 μM in the presence of ferrihydrite, while merely ca. 30 μM was dissolved from goethite. Re-adsorption of Fe^2+^ under these conditions is very low, as shown by previous studies^15,26^, and therefore, these results were a direct reflection of the magnitude of reductive dissolution. In the presence of O_2_, the Fe^2+^ concentrations decreased for both iron oxides (Fig. 4, black lines), and relative to the anaerobic conditions, the effect was largest in the case of goethite. In a previous study, we showed that the re-oxidation of Fe^2+^ in the presence of ferrihydrite was low at pH 4.5^26^, and therefore, the reduced Fe^2+^ concentration indicated an increase in the contribution from the catalytic oxidation of 2,6-DMHQ at the expense of reductive dissolution. Some re-oxidation of Fe^2+^ likely occurred in the presence of goethite, as indicated by the decreases in the concentration after ca. 100 min (Fig. 4b).

The addition of TPA had only small effects on the Fe^2+^ concentrations except under aerobic conditions in the presence of ferrihydrite, where the concentration decreased dramatically in the presence of TPA (Fig. 4a). A similar effect was also observed in the presence of acetate buffer (Fig. S4). The results from the experiment performed in the absence of TPA showed that this decrease in the Fe^2+^ concentration cannot be caused by oxidation via O_2_. Instead, similar effects were observed when 30 μM H_2_O_2_ was added to the aerobic 2,6-DMHQ-ferrihydrite experiment (Fig. 4a). The reason for these low Fe^2+^ concentrations will be further discussed below. Importantly, under anaerobic conditions, the release of Fe^2+^ from ferrihydrite in the absence and presence of TPA was very similar despite the fact that 2,6-DMHQ oxidation was faster when TPA was present (c.f. Figs 3a and 4a). Furthermore, the faster generation of 2,6-dimethoxy-1,4-quinone (2,6-DMBQ (1,4-(CH_3_O)_2_-2,6-C_6_H_2_O_2_)) in the presence of TPA (Fig. S5) implied that for every 2,6-DMHQ molecule oxidized to 2,6-DMBQ via reductive dissolution, the release of Fe^2+^ from ferrihydrite was slowed by the presence of TPA (Fig. S6). This finding is consistent with the effects of electrostatic interactions on the desorption kinetics and the fact that the adsorption of TPA should lower the surface charge, which in turn will slow the desorption of cations, including Fe^2+^ ^44^.

### Generation of hydroxyl radicals

**Figure 5:**
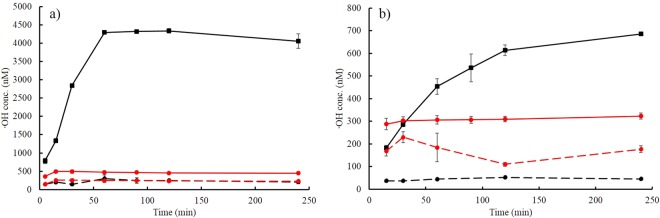
Generation of ·OH from reactions between 90 µM 2,6-DMHQ (0.44 µmol/m^2^) and (**a**) ferrihydrite and (**b**) goethite in the presence of 300 µM TPA. The black and red lines represent pH 4.5 and 7.0, respectively. The experiments were carried out under aerobic conditions (solid lines) and anaerobic conditions (dashed lines). The error bars are standard deviations of triplicate measurements. Some of these error bars are smaller than the size of the symbols and are therefore not distinguishable.

In line with the parallel reductive dissolution and catalytic oxidation processes, which likely produce both Fenton reagents, the reactions between 2,6-DMHQ and the iron oxides generated ·OH, as indicated by the oxidation of the TPA probe (Fig. 5). These results were in general agreement with those of previous investigations of similar reactions in homogeneous solutions. Kerem *et al*. reported the spontaneous production of H_2_O_2_ by the reaction between 2,5-DMHQ and FeCl_3_ in aqueous solution and further observed the cleavage of polyethylene glycol (PEG), which was presumably caused by ·OH^16^. These findings were supported by Jensen *et al*. who detected rapid O_2_ consumption in the same reaction^12^.

**Figure 6:**
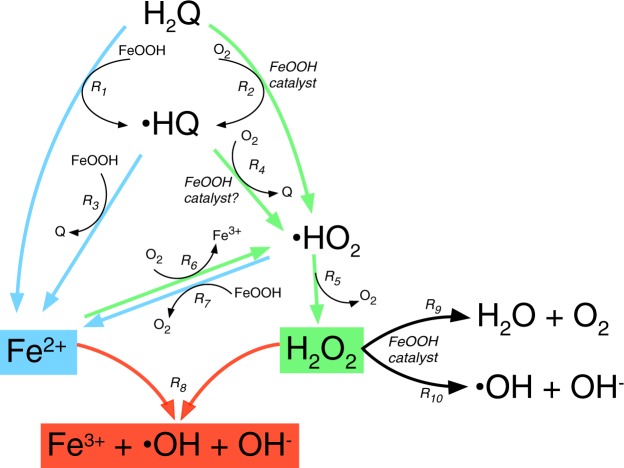
Proposed scheme of the main reactions between 2,6-DMHQ (H_2_Q) and ferrihydrite or goethite (both Fe oxides are denoted FeOOH) that lead to the generation of ·OH. For the sake of clarity, only the generation of H_2_O_2_ via the disproportionation of ·HO_2_ is included, while the reduction of ·HO_2_ by the hydroquinone is omitted.

Hence, the reaction between hydroquinones and Fe^3+^ in both the liquid and solid states generates ·OH, but as our results showed, the amount of ·OH formed via the heterogeneous reaction is strongly dependent on the experimental conditions and the form of solid Fe^3+^ (Fig. 5). These results can be rationalized by considering the one-electron 2,6-DMHQ oxidation reactions of the reductive dissolution and catalytic oxidation processes (Fig. 6). H_2_O_2_ is formed by the disproportionation or reduction (not included in Fig. 6) of superoxide (·HO_2_), which is generated via the reduction of O_2_ by the hydroquinone and semiquinone (reactions R_2_, R_4_ and R_5_ in Fig. 6). Based on these reactions, reducing the O_2_ supply will also reduce the generation of H_2_O_2_, and thus, the very low amounts of ·OH produced under anaerobic conditions in the presence of both ferrihydrite and goethite (Fig. 5) were a direct consequence of the shortage of H_2_O_2_.

On the other hand, the stark contrast in ·OH production by ferrihydrite and goethite of almost an order of magnitude at pH 4.5 under aerobic conditions (Fig. 5) is largely caused by the difference in the contribution of reductive dissolution. The oxidation of 2,6-DMHQ and the generation of Fe^2+^ both showed that the rate and extent of reductive dissolution was far greater in the presence of ferrihydrite than in the presence of goethite (Figs 3 and 4). This difference is in agreement with the higher reduction potential of ferrihydrite compared with that of goethite^45^ and the fact that reductive dissolution by 2,6-DMHQ is effective at much higher dissolved Fe^2+^ concentrations in the presence of ferrihydrite^26^. The initial 2,6-DMHQ oxidation reaction followed approximately first-order kinetics (Fig. S7), and the difference in the first-order rate constants between the aerobic and anaerobic conditions with ferrihydrite was a factor of 1.7, while the corresponding difference for goethite was a factor of 9.0. This difference indicates that the rates of catalytic oxidation and reductive dissolution were much closer for ferrihydrite than for goethite. Thus, not only is more Fe^2+^ generated from ferrihydrite, but also, the timing of the production of Fe^2+^ and H_2_O_2_ seems to be better synchronized, which enhances the efficiency of the Fenton reaction and thereby contributes to the difference between ferrihydrite and goethite in ·OH production.

According to this reasoning, the increase in the rate of reductive ferrihydrite dissolution induced by TPA that was discussed above will favour the Fenton reaction. Although the reductive rate increased, the release rate of Fe^2+^ into solution did not increase (cf. Figs 3a and 4a), implying that during the dissolution process, more Fe^2+^ temporarily accumulated at the ferrihydrite surface in the presence of TPA. This accumulation increases the likelihood of the simultaneous presence of Fe^2+^ and H_2_O_2_ at the surface and further reinforces the efficiency of the Fenton reaction. This enhanced efficiency was also indicated by the decrease in the Fe^2+^ concentration in the aerobic ferrihydrite experiment at pH 4.5, where the generated H_2_O_2_ more effectively oxidized Fe^2+^ in the presence of TPA (Fig. 4a). Moreover, previous studies have shown that the adsorption of oxyanions and low-molecular-weight organic acids inhibits H_2_O_2_ decomposition^46–49^. Hence, by preventing the decomposition of H_2_O_2_ (Fig. 6, reaction R_9_) the adsorption of TPA may further promote the Fenton reaction. Our results show that both the TPA probe and acetate buffer (Fig. S4) influence the redox reactions and ·OH generation. In fact, TPA can be considered a reasonable model of the low-molecular-weight aromatic carboxylic acids present in natural organic matter, and therefore, the results provide some indication of processes that may occur in more complex soil environments.

Finally, the reduction in ·OH production at pH 7.0 was likely caused by several factors. The results of the anaerobic experiments at this pH indicated that reductive dissolution dominated (although some 2,6-DMHQ oxidation might be caused by low concentrations of O_2_), but at the same time, the rate of catalytic oxidation increased substantially, as indicated by the very rapid oxidation of 2,6-DMHQ under aerobic conditions at pH 7.0 (Fig. 3). This increase in rate is a consequence of the increase in the deprotonation of 2,6-DMHQ^17,26^ and shifts both the ferrihydrite and goethite systems towards catalytic oxidation of 2,6-DMHQ. Thus, the low ·OH production at pH 7.0 is likely caused by the limited supply of Fe^2+^, which is further amplified by the rapid re-oxidation of Fe^2+^ at pH 7.0 (Fig. 6, reaction R_6_). In addition, H_2_O_2_ is less stable at pH 7.0 and decomposes into H_2_O and O_2_, a reaction that is catalysed by iron oxide surfaces (Fig. 6, reaction R_9_). Hence, both Fenton reagents are destabilized at pH 7.0.

In a broader sense, the collective results of this study add to the evidence suggesting that the potential toxicity of iron oxide nanoparticles is partly related to their role as nanoreactors that generate ·OH. In this respect, the presented results provide new evidence that both Fenton reagents can be generated from reaction with a biological organic reductant, and no separate source of H_2_O_2_ is needed. The present study, which was conducted at a soil-relevant pH, also supports the idea that extracellular hydroquinones or similar reducing metabolites produced by soil-living fungi can disrupt the associations between soil organic matter and iron oxide via the generation of ·OH and thereby liberate accessible nutrients into the soil environment.

## Materials and Methods

2,6-DMHQ, ferrozine [3-(2-pyridyl)-5,6-bis(4-phenylsulfonic acid)-1,2,4-triazine], and 2-hydroxyterephthalic acid were acquired from Sigma-Aldrich, Sweden. Disodium terephthalate was purchased from Alfa Aesar, Sweden. All chemicals were of pro analysis quality or better, and all solutions were made with ultra-pure water (<2 µS) that was boiled for at least 1 h. After cooling, the water was thoroughly purged with N_2_ for 1 h and finally stored in sealed glass bottles at 4 °C.

Six-line ferrihydrite (Fe_2_O_3_·9H_2_O) was synthesized according to the method of Schwertmann and Cornell^50^. First, 20 g of Fe(NO_3_)_3_·9H_2_O (Merck) was dissolved in 2 L of preheated distilled water under rapid stirring. The solution was kept at 75 °C for 10–12 minutes and then rapidly cooled to room temperature. The obtained suspension was transferred to a dialysis bag and dialyzed until the conductivity was ≤10 µS/cm. The solid concentration of ferrihydrite in the final suspension was 1.7 g/L, and the specific surface area (SSA) was estimated to be ca. 300 m^2^/g^26^. The ferrihydrite used in this study was approximately 1 month old (+/−1 week) unless otherwise stated. The structural identity and morphology of the particles were examined by X-ray diffraction (XRD) and transmission electron microscopy (TEM), respectively (Figs S1 and S2).

Goethite (α-FeOOH) was prepared according to the method of Hiemstra and van Riemsdijk^51^. A solution of 2.5 M NaOH (EKA Chemicals) was slowly added into a solution of 0.5 M Fe(NO_3_)_3_·9H_2_O while stirring and sparging with N_2_ until the pH reached approximately 12. The product was aged at 60 °C for one week and then transferred to a dialysis bag, as above. The solid concentration of goethite in the final suspension was 10.6 g/L. A subsample was freeze dried, and the SSA was determined to be 66.5 ± 0.0.5 m^2^/g by applying the Brunauer−Emmett−Teller (BET) equation^52^ to the N_2_ adsorption data obtained by means of a Micromeritic Gemini VII 2390a instrument. Prior to storage at 4 °C in the dark, the mineral suspensions were thoroughly purged with N_2_ to remove any carbonate species in solution and at the mineral surface. The goethite structure was confirmed by XRD, and the typical needle-shaped morphology was identified by TEM (Figs S1 and S2).

Isotherms of TPA and hTPA adsorption on ferrihydrite were determined from batch experiments. Various volumes of TPA and hTPA stock solutions were added to 115 m^2^/L ferrihydrite in a 0.1 M NaCl medium. Aliquots were filtered through a 0.2 µM syringe filter after 5, 15, 30, 60 and 120 min. TPA was measured spectrophotometrically using a NanoDrop 2000 instrument (Thermo Scientific) at 242 nm, and each data point was an average of five measurements per aliquot. The TPA concentration was calculated from a linear standard (0–500 µM), resulting in a limit of detection (LoD) of 3.8 µM. The hTPA concentration was measured by fluorescence at an excitation wavelength of 315 nm and an emission wavelength of 425 nm using an LS 50 B luminescence spectrometer (Perkin Elmer) with 0.1 M NaCl as the blank. The hTPA concentration was calculated from a linear standard curve (0–500 nM) with a LoD of 0.5 nM. The large difference in the total concentrations of TPA and hTPA explored in this study was motivated by the conditions of the 2,6-DMHQ-iron oxide experiments, which contained ca. 300 μM TPA and 100–6000 nM hTPA.

The adsorption of TPA and hTPA onto ferrihydrite and goethite was also characterized by infrared (IR) spectroscopy using the simultaneous infrared and potentiometric (SIPT) method described by Loring *et al*. and Krumina *et al*.^44,53^. Ferrihydrite and goethite over-layers were prepared on an attenuated total reflectance (ATR) ZnSe crystal by evaporating 0.7 mL of a ferrihydrite (1.7 g/L) or goethite (2.0 g/L) suspension, respectively. The ATR crystal was coupled to a titration vessel and placed inside an evacuated (1.42 mbar) Fourier transform infrared spectrometer (Bruker Vertex 80 v) in a temperature-controlled room at 21 °C. A ferrihydrite or goethite suspension in 0.1 M NaCl medium was added to the titration vessel and slowly stirred. The pH was adjusted to the desired value and kept constant with an automated and computer-controlled burette system (Methrom 907 Titrando and Tiamo 2.4 software) during the experiment. A background spectrum of 4096 scans was collected of the equilibrated over-layer and suspension, after which TPA or hTPA was added. One IR spectrum per min was collected during the first hour of adsorption, and thereafter, the collection time was increased to 512 scans per spectrum (approximately one spectrum per 7.5 min). IR spectra were recorded between 700–4000 cm^−1^ at a resolution of 4 cm^−1^.

The generation of ·OH, Fe^2+^ and hydroquinone oxidation products from the reactions between 2,6-DMHQ and ferrihydrite or goethite nanoparticles was studied in batch experiments at pH values of 4.5 and 7.0 with 50 mL of a 0.1 M NaCl suspension containing 200 m^2^/L iron oxide and 90 µM 2,6-DMHQ in the absence or presence of 300 µM TPA or 30 µM H_2_O_2_. For the experiments in the presence of TPA, TPA was added ca. 60 s before the addition of 2,6-DMHQ and/or H_2_O_2_, and the suspension was thoroughly mixed on an end-over-end rotator. Sample aliquots were removed from the suspension at different time points between 5–240 min. All solutions were adjusted to the required pH with 40 mM HCl or NaOH in 0.1 NaCl medium prior to the experiment (except in one experiment in which the interference from acetate buffer was tested) and during the experiment when needed (see Table S1). For the Fe^2+^ and quinone analyses, 1.3 and 1.0 mL portions of the suspension, respectively, were immediately filtered through a 0.2 µM syringe filter. For ·OH determination, a 1.5 mL aliquot of the suspension was reacted with 56 µL of 16 mM NaH_2_PO_4_ in 0.1 M NaCl for 1 h prior to filtration in order to desorb most of the hTPA (see SI for further details). Analysis of the quinone species, Fe^2+^ and ·OH was conducted immediately after filtration. All experiments were performed in the dark in triplicate. However, analysis of the quinone species by high-performance liquid chromatography (HPLC) was carried out for only one of these replicate time series.

2,6-DMHQ-iron oxide batch experiments were performed at two different oxygen levels, denoted as anaerobic and aerobic in the following text. Prior to the anaerobic experiments, all solutions were sparged with N_2_ for 20–30 minutes, and the experiments were carried out in a glove bag (Sigma-Aldrich) purged with N_2_. This experimental procedure yielded suspensions with an initial [O_2_] = 0–20 µM. The aerobic experiments were conducted under atmospheric conditions with [O_2_] = 270–310 µM in the initial suspensions. The O_2_ concentrations were measured with an Orion Star A213 dissolved oxygen meter (Thermo Scientific).

The 2,6-DMHQ and 2,6-dimethoxybenzoquinone (2,6-DMBQ) concentrations were determined by HPLC. Freshly prepared solutions of 2,6-DMHQ and 2,6-DMBQ were analysed as standards to determine the HPLC response factors. Chromatographic separation was accomplished by a Dionex Ultimate 3000 system (Thermo Scientific) equipped with an RS variable wavelength detector and a 4.6 × 150 mm, 3.5 µm XDB-C18 Eclipse column (Agilent, USA), and this equipment was controlled by Chromelon® software. The column flow rate was 1.0 mL/min, and the solvent system consisted of H_2_O/acetonitrile/formic acid in a 90:10:0.1 ratio. The injection volume was 25 µL, and chromatograms were recorded at wavelengths of 282 and 290 nm for 2,6-DMHQ and 2,6-DMBQ, respectively.

The Fe^2+^ concentration was determined by ferrozine assay^54^. A filtered sample with a volume between 0.5 and 2 mL was mixed with 100 µL of 1% (wt/vol) aqueous ferrozine and diluted to a final volume of 2.3 mL with 0.1 M NaCl or acetate buffer. The absorbance of the solution was measured after 5 min with an Ultrospec 3000 spectrophotometer (Pharmacia Biotech) at 562 nm. Either 0.1 M NaCl or 0.1 M acetate buffer was used as the blank. The Fe^2+^ concentration was determined from a linear standard curve (0–100 µM) made from FeSO_4_. This experimental method resulted in a LoD of 2.0 µM.

The ·OH species was detected via its reaction with TPA and the formation of the hydroxylated product, hTPA^55^. The hTPA concentration was determined by fluorescence spectroscopy as described above, and further experimental details are provided in SI.

## Electronic supplementary material

Supplementary information
