# Supplementary material for: Generation of hydroxyl radicals from reactions between a dimethoxyhydroquinone and iron oxide nanoparticles
Source: Sci Rep. 2018 Jul 17;8:10834. doi: 10.1038/s41598-018-29075-5 (PMC6050337; doi:10.1038/s41598-018-29075-5)
Supplement: Supplementary file 1 — Supplementary information [file 41598_2018_29075_MOESM1_ESM.pdf]

Supporting information

Generation of hydroxyl radicals from reactions between a dimethoxyhydroquinone and iron oxide nanoparticles

Gry Lyngsie,[1] Lelde Krumina,[1,2] Anders Tunlid,[2] Per Persson[1,2]

[1]Centre of Environmental and Climate Research, Lund University, SE-223 62, Lund, Sweden
[2]Department of Biology, Lund University, SE-223 62, Lund, Sweden

Content

S1. Experimental

S2. Results

The supporting information contains 7 figures and 1 table.

S1. Experimental

2,6-Dimethoxy-1,4-hydroquinone (2,6-DMHQ), ferrozine [3-(2-pyridyl)-5,6-bis(4-phenylsulfonic acid)-1,2,4-triazine], and 2-hydroxyterephthalic acid (hTPA) were acquired from Sigma-Aldrich, Sweden. Disodium terephthalate (TPA) was purchased from Alfa Aesar, Sweden. All chemicals were pro analysis or of better quality, and all solution were made with ultra-pure water (< 2 μS) that was boiled for at least 1 h. After cooling it was thoroughly purged with N_2 for 1 h and finally stored in sealed glass bottles at 4 ºC.

S1.1. Mineral characterization

The mineralogy of the dried iron oxide particles was assessed by X-ray diffraction (XRD) analysis on un-oriented samples where a small amount of ground mineral sample was suspended in ethanol and transferred to a silicon sample holder and evaporated prior to analysis. The goethite was measured using a Siemens 5000 equipped with Co-Kα X-ray source, an Fe filter and a diffracted beam monochromator. The diffractogram was recorded from 10 to 90° 2θ using 0.03° 2θ steps and a step speed of 5 s. The ferrihydrite samples were measured on a Bruker D8 equipped with a Cu-Kα X-ray source and a LYNXEYE XE-T detector and the diffractogram were recorded from 10 to 80° 2θ using 0.1° 2θ steps and a step speed of 307 s. Diffraction peak positions were used to calculate d-values for mineral identification. The iron oxide particle morphology and size were analyzed by a transmission electron microscope JEM1230 Jeol limited (Japan).

S1.2. hTPA recovery

To develop an experimental protocol for removal of adsorbed hTPA using phosphate, competitive adsorption experiments between hTPA and phosphate (P_i) were conducted. Suspensions containing hTPA at total concentrations of 15 nmol/m^2 and 5 nmol/m^2 in presence of ferrihydrite and goethite, respectively, were reacted for 30 min. 56 μL of 16 mM NaH_2PO_4, corresponding to 3 μmol P_i/m^2, was subsequently added and aliquots were extracted and filtered through a 0.2 μm syringe filter after 15, 30, 45 and 60 minutes and hTPA was measured according to the procedure described in the main text.

S1.3. Generation of Fe^{2+} in acetate buffer

The generation of Fe^{2+} from reactions between 2,6-DMHQ and ferrihydrite nanoparticles was studied in batch experiments at pH 4.5. 50 mL 0.1 M acetate buffer suspensions containing

200 m^2/L ferrihydrite and 90 μM 2,6-DMHQ. Sample aliquots were obtained from the suspension at different time points between 5-240 min. For the Fe^{2+} analysis 1.3 mL of the suspension was instantly filtered through a 0.2 μM syringe filter, and subjected to the ferrozine method as described in the main text. The experiments were performed in the dark and carried out in triplicates.

S2 Results

S2.1. Mineral characterization

The X-ray diffractogram of approximately 1 month old ferrihydrite displayed 6 broad peaks at 1.48, 1.51, 1.70, 1.96, 2.21, and 2.50 Å (Figure S1), characteristic of 6-line ferrihydrite, and the TEM revealed spherical particles with an average particle size ca. 4–5 nm (Figure S2), which is in agreement with previous results.[1] These data were compared to ferrihydrite aged for ~2 years, and the aged sample displayed additional broad peaks at 1,71 and 4.18 Å (53 and 21˚2θ), indicating partial transformation into goethite nano-particles. It is notable though that still the 6-line ferrihydrite dominated the X-ray diffractogram after 2 years of aging at 4 ºC in the dark. The XRD analysis of the goethite sample displayed distinct sharp and well-defined peaks confirming the structural identity (Figure S1). Furthermore, the TEM images displayed goethite's characteristic needle shape morphology and with an estimated length of a few hundred nm and width of 10-20 nm (Figure S2).

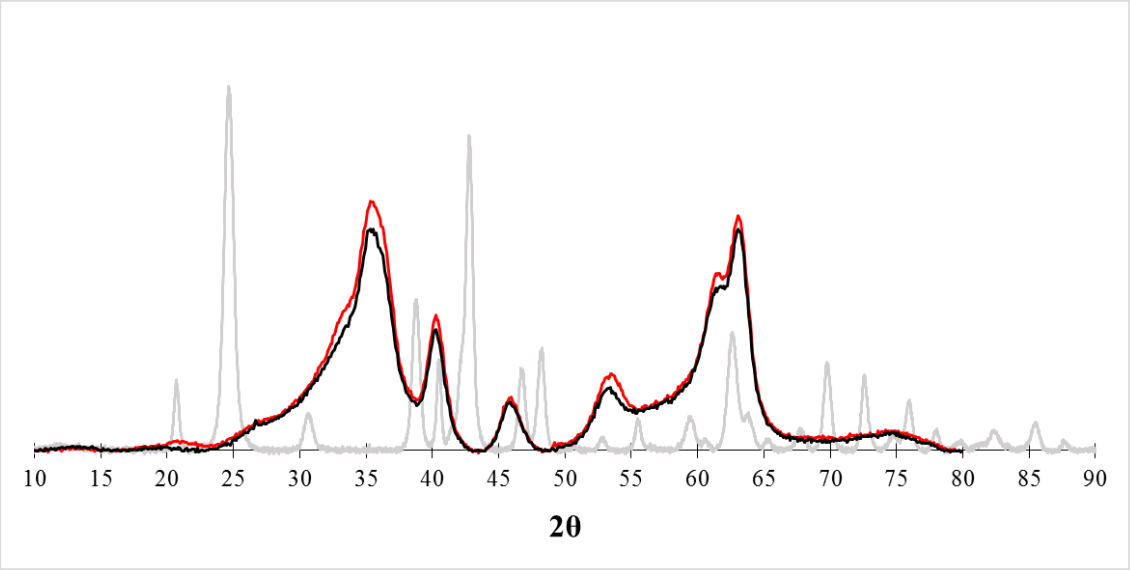

Figure S1 XRD diffractograms of goethite (gray), 1-month-old ferrihydrite (black) and 2-year-old ferrihydrite (red).

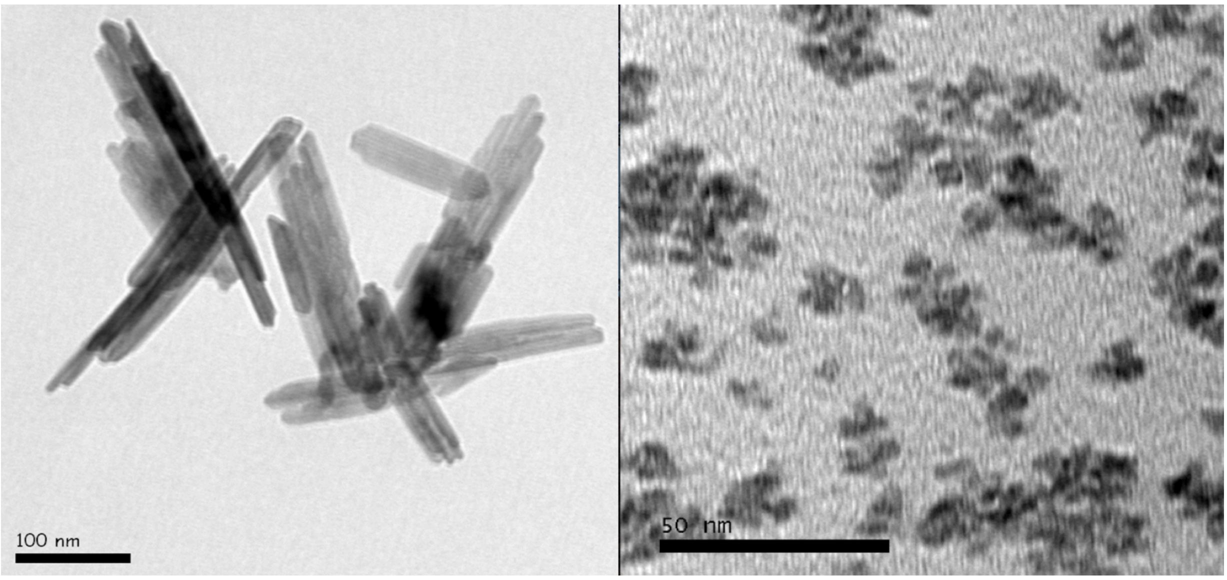

Figure S2. TEM images of goethite (left) and ferrihydrite (right).

S2.2. hTPA recovery

Experiments were conducted to investigate whether phosphate was capable of desorbing pre-adsorbed hTPA from ferrihydrite and goethite surfaces. After 1 h of reaction with phosphate, approximately 80% of the hTPA was recovered from both the ferrihydrite and goethite surfaces at pH 4.5 and ca. 90% for ferrihydrite at pH 7.0 (Figure S2). These results showed that phosphate could be used to recover a major part of the adsorbed hTPA and that this is a necessary procedure to add to the experimental protocol in order to determine ·OH in the presence of iron oxide particles by means of the TPA. With longer reaction times the hTPA recoveries from phosphate-promoted desorption will improve. However, since TPA cannot be used for absolute quantification of ·OH because of the unknown efficiency of the oxidative reaction, we have limited the desorption step in our protocol to 1 h.

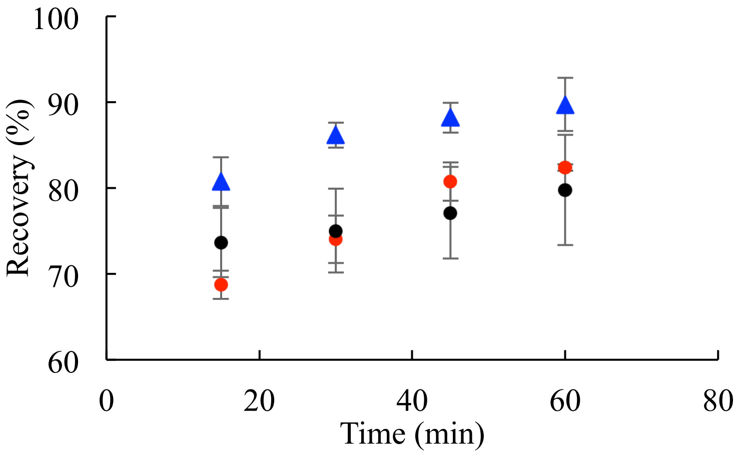

Figure S3. Recovery of hTPA from iron oxides surfaces at pH 4.5 ferrihydrite (black) and goethite (red), and at pH 7.0 ferrihydrite (blue). The hTPA surface coverage prior to the phosphate addition at 3 μmol/m^2 was 15 nmol/m^2 and 5 nmol /m^2 for ferrihydrite and goethite, respectively. The pre-adsorption of hTPA was conducted for 30 minutes and during this time >99 % of the added hTPA adsorbed. Error bars are standard deviations of triplicates.

S2.3. Complementary figures

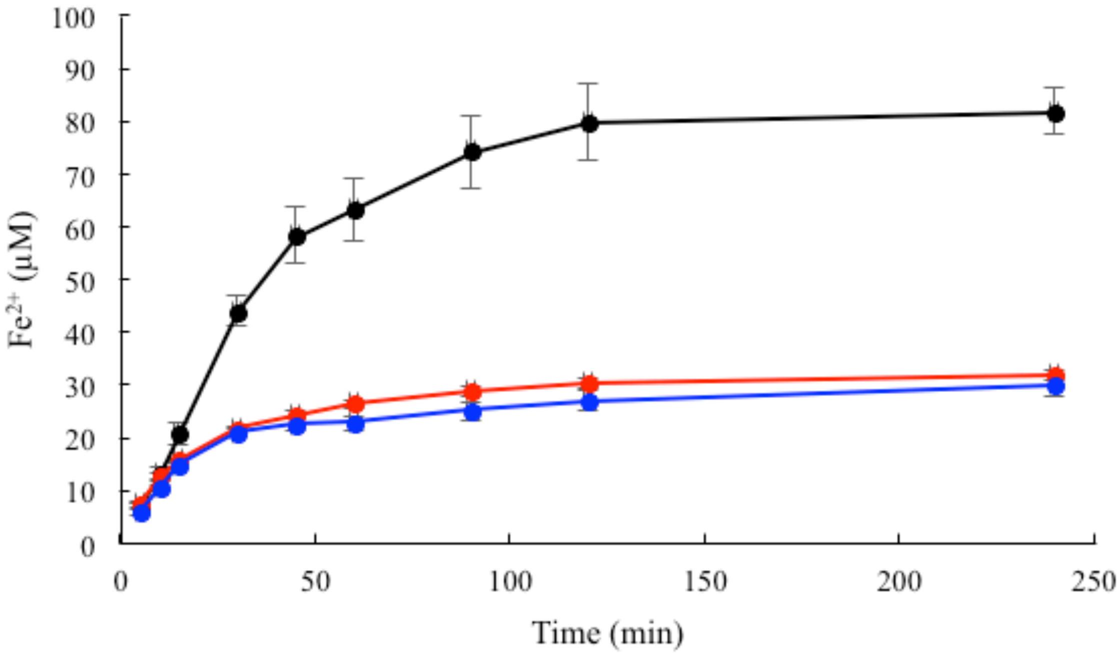

Figure S4. Generation of Fe^{2+} from reactions between 90 μM 2,6-DMHQ (0.44 μmol/m^2) and ferrihydrite in 0.1 M NaCl (black), 0.1 M acetate buffer (red) and 0.1 M NaCl and 300 μM TPA (blue) at pH 4.5 under aerobic conditions. Note that the ferrihydrite used here was older

than the ferrihydrite used in the main text, and cannot be compared directly. Error bars are standard deviations of triplicates.

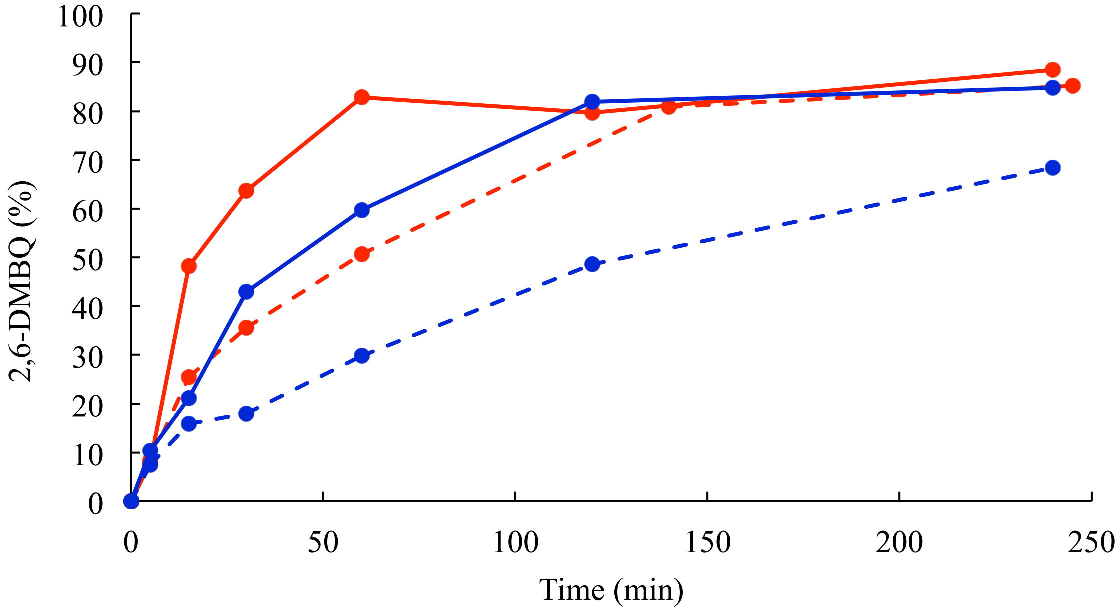

Figure S5. Generation of 2,6-DMBQ from reactions between 90 μM 2,6-DMHQ (0.44 μmol/m^2) and ferrihydrite at pH 4.5 as a function of time at aerobic (red) and anaerobic (blue) conditions. Experiments were performed in absence (dashed line) and presence (solid line) of 300 μM TPA.

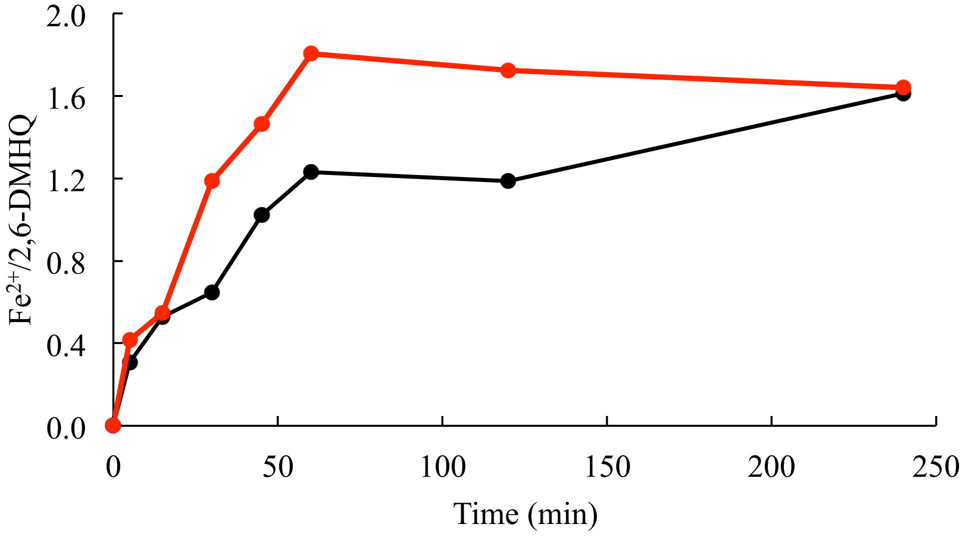

Figure S6. Fe^{2+}/2,6-DMHQ ratios obtained during reactions between 90 μM 2,6-DMHQ (0.44 μmol/m^2) and ferrihydrite under anaerobic condition at pH 4.5. Red and black lines represent experiments in presence and absence of TPA, respectively.

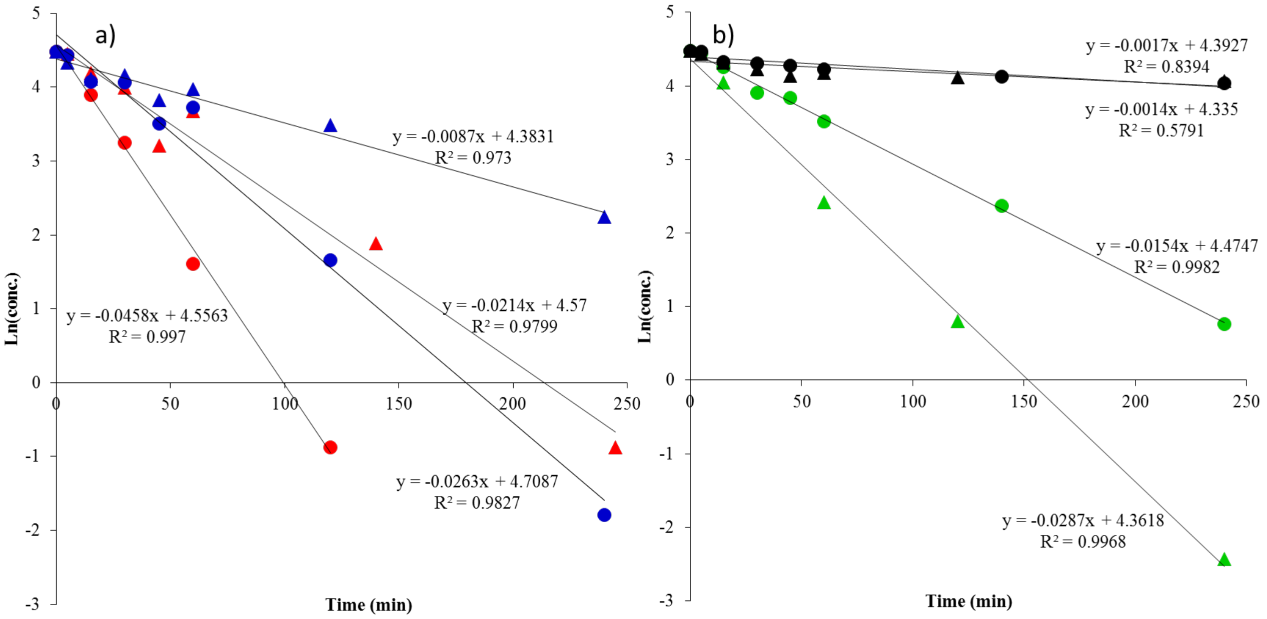

Figure S7. First-order kinetics of 90 μM DMHQ oxidation in the presences of a) ferrihydrite and b) goethite under anaerobic (blue and black) or aerobic (red and green) conditions. Experiments were performed in presence (circles) and absence (triangles) of 300 μM TPA.

S2.4. Complementary table

Table S1. Total amount of acid added to the batch experiments.

	Sample	pH (±0.1)	Acid additions (mmol)*
Aerobic	Ferrihydrite	4.5	0.008
	Ferrihydrite with TPA	4.5	0.006
	Ferrihydrite	7.0	-
	Ferrihydrite with TPA	7.0	-
Anaerobic	Ferrihydrite	4.5	0.006
	Ferrihydrite with TPA	4.5	0.004
	Ferrihydrite	7.0	-
	Ferrihydrite with TPA	7.0	-
Aerobic	Goethite	4.5	0.002
	Goethite with TPA	4.5	0.001
	Goethite	7.0	-
	Goethite with TPA	7.0	-
Anaerobic	Goethite	4.5	0.002
	Goethite with TPA	4.5	-
	Goethite	7.0	-
	Goethite with TPA	7.0	

*Calculated from the HCl concentration of 40.0 mM and drop-wise volume additions corresponding 50 μL/drop.

Reference

1 U. Schwertmann and R. M. Cornell, *Iron Oxides in the Laboratory: Preparation and Characterization*, Wiley-VCH, Weinheim, 2000.
